# Influence of mental health conditions and symptoms on contraceptive use among adolescent girls and young women: a scoping review

**DOI:** 10.3389/frph.2025.1620736

**Published:** 2025-09-02

**Authors:** Muziwandile Qiniso Luthuli, Busisiwe Nkala-Dlamini, Nomfundo Nzuza-Moroe

**Affiliations:** ^1^ Department of Social Work, School of Human and Community Development, University of the Witwatersrand, Johannesburg, South Africa; ^2^ Department of Speech Pathology and Audiology, School of Human and Community Development, University of the Witwatersrand, Johannesburg, South Africa

**Keywords:** mental health, mental disorders, contraception, unintended pregnancy, adolescents, young women, hormonal contraceptive use

## Abstract

**Background:**

Adolescent girls and young women (AGYW) across the globe face a high risk of mental health challenges and unintended pregnancies, which are often complex and intertwined within their psychosocial environments. While earlier studies have examined contraceptive use, the influence of mental health conditions and symptoms on a broader range of contraceptive options among AGYW has not been thoroughly investigated.

**Objectives:**

This scoping review aims to map and synthesize peer-reviewed literature to understand how mental health conditions and symptoms influence contraceptive use among AGYW globally.

**Methods:**

This review followed Arksey and O'Malley's framework. Databases searched included PubMed, MEDLINE, PsycINFO, CINAHL, Psychology and Behavioral Sciences Collection, Web of Science, and African Journals Online. We included studies on AGYW aged 10–25 years that addressed how mental health conditions and symptoms influence contraceptive use.

**Results:**

Of the 9,817 records identified, only 17 studies met inclusion criteria. Depression (65%; *n* = 11) was the most frequently studied mental health factor, followed by stress (29%; *n* = 5), proxies for mental distress (24%; *n* = 4, including antidepressants and psychotropic drug use), and anxiety (18%; *n* = 3). Hormonal contraceptives (HCs) (100%; *n* = 17) were the most studied methods. Among the included studies, 71% (*n* = 12) found that mental health conditions and symptoms, such as depression, anxiety, psychological distress, stress, antidepressants, and psychotropic drug use, were associated with lower odds of HC use. Conversely, 29% (*n* = 5) found these factors to be associated with higher odds of HC use, particularly oral and non-oral methods. Stress and depression were associated with lower odds of consistent contraceptive use, while anxiety demonstrated mixed effects. Two studies reported no association between mental health factors and contraceptive use. Eleven studies focused on adolescents and six on young adults, showing a bidirectional influence between mental health and contraceptive use, with inconsistent findings across age groups.

**Conclusions:**

Mental health factors affect contraceptive use in different ways. Depression and stress reduce the odds of consistent HC use, especially among non–oral contraceptive users, while anxiety shows mixed outcomes. Findings underscore the complex influence of mental health factors on contraceptive behavior among AGYW and highlight the need for age–specific policies and interventions tailored to this high–priority population.

## 1 Introduction

Global and regional evidence points to a growing concern about the complex relationship between mental health factors and contraceptive use among adolescent girls and young women (AGYW) (1, 2). Mental distress in this population is on a constant rise and often intersects with reproductive health vulnerabilities. For instance, in a recent large-scale study conducted in the United States, over one-third (35%) of young women reported delays in accessing contraception, with depressive symptoms, anxiety, and stress identified as key contributing factors (3). These findings reflect broader global trends. In 2019, among the estimated 300 million adolescents aged 15–19 years globally, only 29.8 million reported using any form of contraception (4). These figures demonstrate both the scale of contraceptive needs among AGYW and the importance of understanding how mental health factors may influence contraceptive behaviors. This low uptake of contraception partly contributes to the high rates of unintended pregnancies among AGYW, which remain a major public health concern, especially in low- and middle-income countries (LMICs) (5, 6).

Each year, an estimated 21 million pregnancies occur among adolescents aged 15–19 years, and nearly half are unintended (6, 7). Unintended pregnancy among adolescents results in about 12 million births every year in developing countries (6), hence the need for effective interventions to address the associated health, educational, and socioeconomic risks (8). More specifically, unsafe abortions contribute 5.5% of unplanned pregnancies in adolescents, which reflects a pressing need for integrated sexual and reproductive health (SRH) strategies to protect the well-being of this vulnerable population group (7). Globally, contraceptive use among AGYW is shaped by a combination of psychological, contextual, and behavioral factors (9–11). The relationship between mental health factors and adolescent pregnancy is complex and multifaceted. Mental health disorders, such as depression and anxiety, may function both as a precursor to and outcome of unintended pregnancy among AGYW (12). A longitudinal study in Australia showed a significant shift in contraceptive preferences among young women, with the use of contraceptive pills declining from 60%–41% over five years, while long-acting reversible contraceptive (LARC) use increased from 13%–21% (13). This change may be due to growing awareness of LARC effectiveness, evolving attitudes towards daily pill adherence, concerns about hormonal side effects, or policy initiatives promoting LARC use (14–16).

Moreover, in the United States, college students with a history of depressive disorders were found to be significantly less likely to use dual contraceptive methods compared to other college students without a history of depressive symptoms (17). These findings indicate that mental health conditions may impact contraceptive choice and use, underscoring the need for integrated mental health and SRH services. However, global evidence on the relationship between mental health factors and contraceptive use remains mixed and, at times, contradictory. A recent systematic review by Kraft et al. (18) concluded that most studies did not find a statistically significant association between oral contraceptives (OCs) and mental health symptoms. Nonetheless, some studies reported that adolescents may be more vulnerable to depressive symptoms when using specific hormonal contraceptives (HCs) (19, 20). Psychological distress has also been associated with lower use of OCs and a greater preference for condoms or LARCs, and in some cases, complete contraceptive non-use (13). While some AGYW may benefit from the mood stabilizing effects of combined hormonal contraceptives (CHCs), others, particularly those with pre-existing mental health conditions, may experience increased emotional instability or higher antidepressant use (21). These findings underscore the importance of personalized contraceptive counseling, especially for individuals taking psychotropic medications or those at risk of inconsistent contraceptive use (18, 22).

In LMICs, and particularly in sub-Saharan Africa, the relationship between mental health factors and contraceptive use is further complicated by socio-economic inequalities and limited access to integrated SRH services (23, 24). Research from sub–Saharan Africa indicates that approximately 27% of adolescents experience depression, 30% suffer from anxiety, and 41% face mood or behavioral issues (25). Another study conducted in Ethiopia found that poor mental health has been associated with increased unmet need for contraception among AGYW (25). In 2023, a national survey was conducted in South Africa among university students who reported a 30-day prevalence of 37.1% for anxiety and 16.3% for mood disorders (26), highlighting the high burden of mental health issues within this population group. The longitudinal data also revealed that the prevalence of common mental disorders rises sharply with age, increasing from 10.1% in 13-year-olds to 33.1% in 22-year-olds among AGYW from rural settings (27). These patterns are deeply influenced by broader structural and systemic factors, including but not limited to poverty, gender-based violence, and limited access to quality mental health and SRH services.

Moreover, these psychosocial vulnerabilities may be further worsened by HC use, with several studies suggesting that certain HCs can trigger or intensify depressive and anxiety symptoms among adolescents (28–30). Although some populations may benefit from the mood protective effects of combined oral contraceptives (COCs) (31, 32), other studies have also suggested that adolescent users of HCs, especially those using progestin-only methods, are at high risk of depressive symptoms and antidepressant use (33, 34). Skovlund et al. (30) and Lundin et al. (35) found that adolescents who were LARC users, such as those who used the levonorgestrel intrauterine device (LNG-IUD) method, were prone to a higher risk of experiencing depression or anxiety, although this association was not consistently observed across studies (36). While a significant body of research that explores the relationship between mental health factors and contraceptive use is available, the evidence is dispersed and often lacks a specific focus on AGYW, particularly in LMICs. For instance, while some studies suggest that HC use during adolescence may increase the risk of major depressive disorder (MDD) later in life (28), the evidence on the impact of HCs on mood symptoms remains inconsistent and inconclusive, as reported in several prior studies (37–39). Moreover, existing evidence has not thoroughly explored how mental health factors influence contraceptive behavior across regions and countries in the Global South, thereby compounding the persistent challenge of unintended pregnancies among AGYW (40). Additionally, some studies concentrate on adult women, with the adolescent population either being underrepresented or entirely overlooked (21, 41). Given the breadth, conceptual complexity of the topic, and potential methodological skewness in the scope of existing literature, including variations in study findings and populations, a scoping review was deemed to be the most appropriate approach to adopt. This approach enables mapping key concepts, synthesizing diverse forms of evidence, and identifying knowledge gaps in areas where literature is either emerging, lacks conceptual clarity, or is fragmented (42).

Therefore, to explore and identify critical gaps, this scoping review aimed to map and synthesize the available peer-reviewed literature to examine the influence of mental health conditions and symptoms on contraceptive use among AGYW globally. The findings of this study provide valuable insights that can inform and strengthen public health policies and interventions aimed at addressing unique mental health and SRH challenges encountered by AGYW worldwide.

## 2 Materials and methods

### 2.1 Design

This is a scoping review of published peer–reviewed literature that examined the influence of mental health conditions and symptoms on contraceptive use among adolescent girls and young women aged 10–25 years. This review serves as a precursor to a larger doctoral research project investigating the psychosocial factors and sexual gender norms as correlates of adolescents' intentions to use dual contraceptive methods in South Africa. The scoping review included peer–reviewed studies published between 2012 and 2024. Additionally, the review was conducted strictly in adherence to the guidelines of the Preferred Reporting Items for Systematic Reviews and Meta–Analyses–extension for Scoping Reviews (PRISMA–ScR) (43, 44) and was grounded in Arksey and O'Malley's methodological framework for scoping studies (42). The PRISMA–ScR Checklist in line with this review is available in (Supplementary Appendix 1). The framework developed by Arksey and O'Malley was utilized due to its systematic and adaptable methodology, which facilitates the mapping of key concepts and the identification of research gaps within a wide array of diverse literature (42). The five stages of this framework were applied and noted as follows: i. formulating the research question, ii. identifying relevant studies, iii. selecting studies for inclusion, iv. extracting and charting the data, and v. collating, summarizing, and reporting the results (42). This iterative process enabled a comprehensive analysis of the influence of mental health conditions and symptoms on contraceptive use by highlighting patterns and trends, while identifying areas for further investigation.

#### 2.1.1 Formulating the research question

The current review was guided by the following broad research question: How do mental health conditions and symptoms influence contraceptive use among adolescent girls and young women globally? This question addresses the need for a broader and more nuanced understanding of how clinical and subclinical mental health factors, whether diagnosed, underdiagnosed, or underrecognized, influence contraceptive behaviors in these vulnerable groups. Adolescents and young adults face barriers to SRH services and, hence, are prone to the risk of unintended pregnancies and sexually transmitted infections (STIs), especially in LMICs (6, 45, 46). Understanding the role of mental health factors can help design effective interventions to improve contraceptive uptake and use in these key segments of the population. A central focus on mental health support and service delivery may empower AGYW to make informed reproductive health decisions, thereby improving both the uptake and consistent use of contraceptive methods across various regions.

#### 2.1.2 Eligibility criteria and identifying relevant studies

In answering the research question, the primary reviewer (M.Q.L) conducted the initial database search between July 2024 and August 2024. The review included eligible peer–reviewed studies that: i. focused on adolescent girls and young women aged 10–25 years; ii. examined the influence or effect of mental health factors such as depression, stress, anxiety, or other relevant mental health conditions on contraceptive use; and iii. included populations from any geographic regions worldwide. The eligibility criteria were determined using the Population–Concept–Context (PCC) framework, as recommended by the Joanna Briggs Institute for scoping reviews (47). Table 1 shows how the PCC framework was applied to conceptualize a review scope and research question.

**Table 1:** PCC framework.

PCC Framework	
(P) Population	Adolescent girls and young women aged 10–25 years, as defined by WHO (6).
(C) Concept	The influence of mental health factors, such as depressive symptoms, major depressive disorder, stress, anxiety symptoms and disorders, mood, and other mental disorders, on contraceptive use.
(C) Context	Global context—encompassing diverse geographic locations and populations.

**Figure 1:**
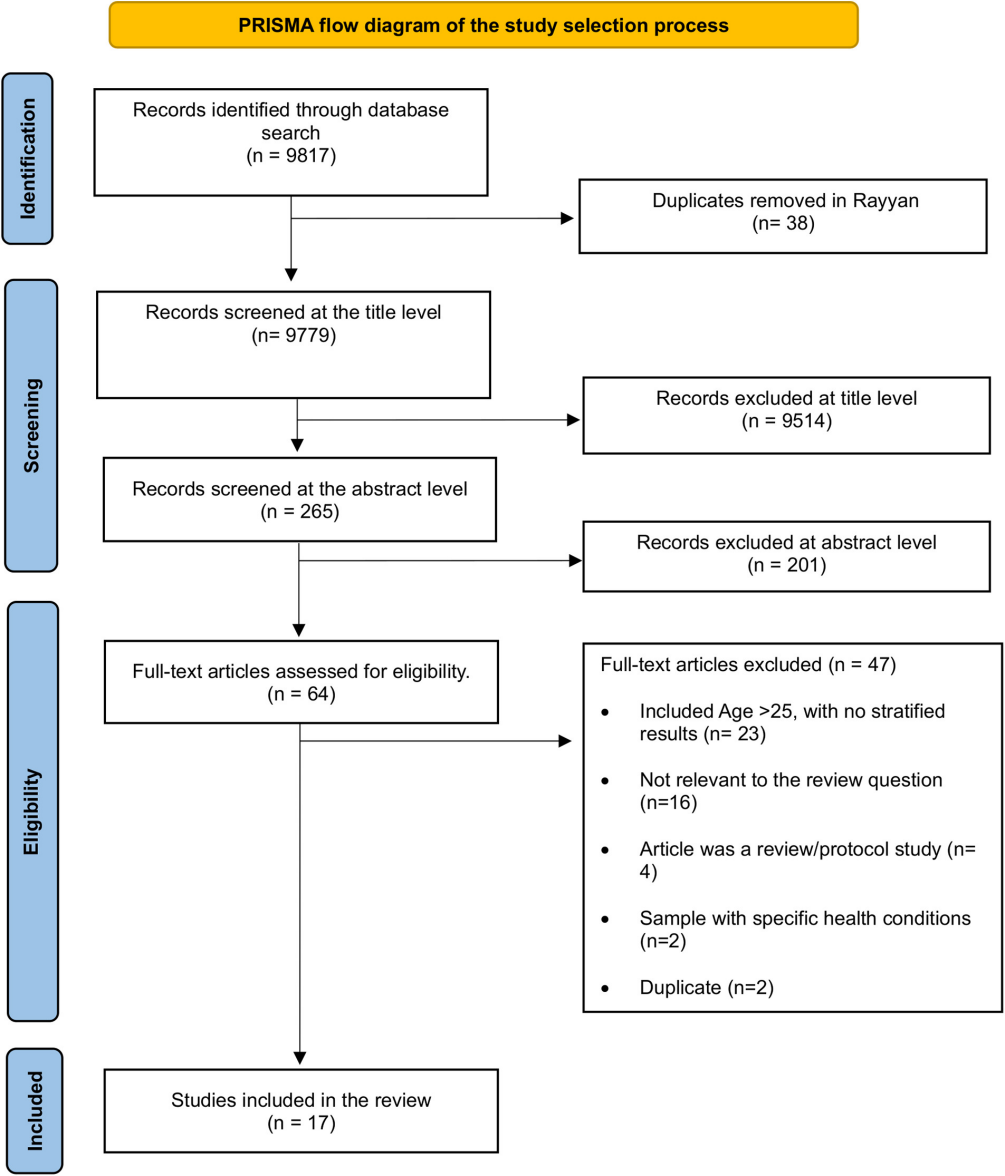
PRISMA flow diagram of the study selection process (81).

The databases that were searched for articles meeting the eligibility criteria included PubMed, MEDLINE with Full Text via EBSCOhost, PsycINFO via EBSCOhost, CINAHL Ultimate with Full Text via EBSCOhost, Psychology and Behavioral Sciences Collection with Full Text via EBSCOhost, Web of Science, and African Journals Online. The search process followed the PRISMA guidelines shown in Figure 1 to ensure transparency and rigor (43). Eligible articles were limited to studies published in English. The language restriction was applied to save time and counteract limited financial resources, as translating non-English publications would have required additional services (48).

The search strategy incorporated a comprehensive set of keywords to ensure broad coverage. The primary search terms included [(female adolescents OR adolescent girls OR teenagers OR young adults) AND (mental health OR mental disorders OR mood disorders OR mental distress OR mental wellbeing)] AND (dual contraceptives OR combined contraceptive methods OR barrier contraception OR barrier birth control OR contraceptive non-use OR condom non-use). Boolean operators “AND” and “OR” were used to combine and separate keywords, allowing for flexibility in identifying relevant studies. Additionally, Medical Subject Headings (MeSH) terms were included to refine the search and enhance precision.

The primary reviewer, M.Q.L, used the library account of the University of the Witwatersrand to access the databases specified and search for relevant studies. Upon retrieving the relevant records, the reviewer utilized a BIB file format to efficiently export references from databases for better management. To ensure the thoroughness of the process of records identification and screening phases, the authors developed a clear inclusion criterion for eligible studies. Additionally, another reviewer, N.M, verified the databases through which the records were identified.

#### 2.1.3 Selecting relevant studies

The primary reviewer (M.Q.L) imported the records that were identified through the database search into the latest version of the Rayyan Systematic Review Software (49). This software was utilized to automatically identify and manually remove duplicates meticulously from the database of records that were imported. The review process involved three stages, namely title screening, abstract screening, and full-text review. Through a database search, we initially identified a total of 9,817 records. After importing them into Rayyan, 38 duplicates were manually identified and removed. We remained with 9,779 records for title screening, and this step excluded 9,514 records, with 265 remaining for the abstract screening stage. After abstract screening, 201 records were excluded, and 64 qualified for the full-text review phase, which finally led to 17 eligible studies.

Moreover, we excluded studies with participants older than 25 years if age-stratified results for the eligible group were not provided, even when the studies were otherwise relevant to the review objective. We also excluded articles that did not match our review question, for example some studies focused on the health-related quality of life (50, 51), intimate partner violence (52, 53), suicidal ideation (54) as predictors, and focused on contraceptive choice (55) or decision making (56), instead of actual use. Review articles and protocol studies were also excluded. Articles with participants who had specific conditions such as attention-deficit/hyperactivity disorder (ADHD) (57) and premenopausal disorder (58) were excluded because these conditions may independently influence mood, cognition, and behavioral regulations, making it difficult to isolate the effects of contraceptive use. Therefore, including such studies could distort the aim of the review, as it would be unclear whether the mental health conditions or symptoms are due to contraceptive use or simply part of temporary gynecological and long-term neurodevelopmental conditions.

Reviewers M.Q.L, N.M, and B.N.D conducted the review process from record screening through to the inclusion stage. The review was carried out independently at each stage of the process. In cases where conflicts arose at any given stage, a third reviewer with a neutral perspective was involved to help resolve disagreements after the discussions in meetings. For instance, each reviewer first worked independently, but when we did not agree on whether a certain article met the inclusion criteria, we held virtual meetings to openly share our reasons for each decision. If we still could not reach an agreement, we consulted a third reviewer who had not been involved earlier to provide an impartial perspective and help us make a final decision about the article. This iterative and collaborative approach ensured that the review process was a systematic, rigorous, and thorough process that met the scientific standards.

#### 2.1.4 Extracting and charting the data

The primary reviewer developed a data extraction workbook in Microsoft Excel to systematically extract data from each included study. The extracted variables included author(s), publication year, study aim, study type and design, study setting, participants and target age groups, sample size, mental health variables, contraceptive methods and types, and a summary of results. The mental health conditions and symptoms extracted from the eligible studies encompass several categories, including proxies for mental distress (e.g., psychotropic drug use and use of antidepressants); depression-related variables (e.g., depression, major depressive disorder, depressive symptoms, depressed mood); anxiety related variables (e.g., anxiety, anxiety symptoms, anxiety disorder); stress and psychological distress (e.g., stress, stress symptoms, psychological distress, perceived stress); and other mental health problems (e.g., personality disorders, intentional self-harm, strength and difficulties).

The review was broadly inclusive of the range of contraceptive methods and types extracted, including HCs, namely oral contraceptives (OCs), which encompassed combined oral contraceptives (COCs), progestin-only pills (POPs), and oral contraceptive pills (OCPs). Non-oral hormonal contraceptives included transdermal patch, vaginal ring, and long-acting methods (LARCs) such as LNG-IUD, injectables, and implants. Additionally, dual contraceptive methods encompassed the use of both HC and barrier methods, such as condoms and diaphragms. Other contraceptive methods included natural family planning, emergency contraception, and withdrawal (Coitus Interruptus) (see Table 2). The data extraction spreadsheet was directed and used to process the relevant information from each of the studies included in the review. All variables in the spreadsheet were carefully selected to address the research question. Prior to the full data extraction process, the data extraction tool was pilot tested by two independent reviewers using three of the included studies. Each reviewer extracted data from the same studies separately to check whether the tool was clear, complete, and able to capture the key information needed to address the scoping review question. Afterwards, a virtual meeting was held to compare the extraction results, resolve inconsistencies, and make minor adjustments to improve the tool based on the discussion. Following this, a primary reviewer conducted a full data extraction, and the extracted data were reviewed by two additional reviewers, B.H.D. and N.M. In addition, together, the reviewers discussed, refined, and reached an agreement on the final data charting and interpretation.

**Table 2:** Study characteristics (*N* = 17).

Author (s), (year)	Study aim	Study approach and design	Study setting (country)	Participants and target age group	Sample size (*N*)	Mental health factors	Contraceptive types	Summary of results
Zettermark et al. (59)	To investigate whether use of hormonal contraception (HC) was associated with adverse psychological health outcomes, and whether this association was modified by age.	Nationwide, quantitative, retrospective cohort study.	Sweden	AGYW aged 12–30 years, with age-stratified results for 12–14, 15–17, 18–20, 21–25, and 26–30 years.	815,662	Psychotropic drugs use as a proxy for psychological distress	Hormonal contraceptives (HCs)—Oral vs. non-oral forms, CHC vs. progesterone-only methods.	• The HC use was strongly associated with the use of psychotropic medication in 12–14-year-old girls, with an odds ratio of 3.46 (95% CI: 3.04 to 3.94). In adult women, there was no association. • The strongest association was with 12–14-year-old users of non-oral progesterone methods, i.e., patches or rings, with an odds ratio of 4.47 (95% CI: 2.08 to 8.78). • Non-oral and progestin-only HC methods had higher odds ratios [OR of 2.27 (95% CI: 1.85 to 2.79)] for psychotropic drug use compared to oral and combined HC methods, even among 15–17-year-olds [OR of 1.52 (95% CI: 1.41 to 1.64)].
Anderl et al. (60)	To examine the association between adolescent oral contraceptive (OC) use and subsequent major depressive disorder (MDD) in early adulthood, analyzing all theoretically justifiable models.	A quantitative, prospective, population-based longitudinal, cohort study.	Netherlands	AGYW aged 13–25 years.	725	Major depressive disorder (MDD)	Oral contraceptives (OCs)	• Adolescent oral contraceptive use increased the risk of major depressive disorder (MDD) in early adulthood (ages 20–25 years) by 41%, [OR of 1.41 (95% CI: 1.08 to 2.18), *p* < .001] with a 72% higher risk in women without a prior MDD history [OR of 1.72 (95% CI: 1.21 to 2.18), *p* < .001]. • Adolescent OC use was associated with a small but robust increased risk for experiencing an episode of MDD, especially among women with no history of MDD in adolescence.
Doornweerd et al. (61)	To examine whether oral contraceptive (OC) use is associated with depressive and anxiety symptoms trajectories from early adolescence into early adulthood, and to explore the effect of age of OC onset on these symptom trajectories.	A quantitative, prospective, longitudinal, cohort study.	Netherlands	AGYW aged 13–24 years.	178	Depressive symptoms and anxiety symptoms.	Oral contraceptives (OCs)	• Depressive symptoms showed an overall increase in late adolescence (> 16 years) with a significant main effect of slope 2 (ß = 1.11, *p* = 0.003), and the interaction effect of slope 2 (> 16 years) and OC use was statistically significant (ß = −1.25, *p* < 0.001). • For anxiety symptoms, only the interaction effect between slope 2 (>16 years) and OC use was significant (ß = −0.74, *p* = 0.023). • Again, an increase of depressive and anxiety symptoms toward the end of adolescence and young adulthood was seen only for non-users of OCs, whereas users maintained stable levels of symptomology.
Stenhammar et al. (62)	To determine which women were potentially at risk of depression in association with LNG-IUD use.	Nationwide, quantitative, prospective, longitudinal cohort study	Sweden	AGYW aged 15–24 years	703,157	Depression	LNG-IUD	• LNG-IUD use was associated with 57% increased risk of depression [AHR 1.57 (95% CI 1.51–1.64)]. compared to non-users and 52% compared to other HC users. • The risk of developing depression was significantly higher if the LNG-IUD was inserted before twenty years of age [AHR 2.57, (95% CI 2.36–2.80)] in comparison to women 20–24 years of age [AHR 1.57, (95% CI 1.49–1.65)], with non-overlapping confidence intervals. • The risk of developing depression was higher for women using the LNG-IUD as their first ever HCs, compared to those with prior HC use [AHR 1.63, (95% CI 1.50–1.78)].
Hall et al. (63)	To prospectively examine the influence of young women's depression and stress symptoms on their weekly consistency of contraceptive method use.	A quantitative, prospective longitudinal cohort study	United States	Young women aged 18–20 years.	689	Depression and stress	Long-acting methods (injectable, LNG-IUD, implant), OCs, ring, patch, and barrier methods (condoms)	• Women with moderate to severe depression and stress symptoms showed significantly lower rates of consistent contraceptive use, with 47% and 69% lower odds each week, respectively (OR 0.53, CI 0.31–0.91 and OR 0.31, CI 0.18–0.52). • Stress was associated with inconsistent use of OCs (OR 0.27, CI 0.12–0.58), condoms (OR 0.40, CI 0.23–0.69), and withdrawal (OR 0.12, CI 0.03–0.50).
Skovlund et al. (30)	To investigate whether the use of specific types of hormonal contraceptives was associated with the risk for first use of antidepressants and first diagnosis of depression at a psychiatric hospital.	Nationwide, quantiative, prospective, longitudinal, cohort study.	Denmark	AGYW aged 15–34 years, with age-stratified results for 15–19, 20–24, 25–29 and 30–34years.	1,061,997	Use of antidepressants as a proxy for diagnosis of depression	Combined oral contraceptives (COCs), progestin-only pills (POPs), transdermal patch, vaginal ring, and IUD.	• Users of COCs had a 23% increased risk of first antidepressant use compared to non-users [RR 1.23 (95%CI, 1.22–1.25)], with higher risks for other methods: 34% for POPs [RR 1.34 (95%CI, 1.27–1.40)], 100% for patches [RR 2.0 (95%CI, 1.76–2.18)], 60% for vaginal rings [RR 1.6 (95%CI, 1.55–1.69)], and 40% for LNG-IUDs (RR 1.4 (95%CI, 1.31–1.42). • Among adolescents aged 15–19 years, risks were even higher by 80% for COCs [RR of 1.8 (95%CI, 1.75–1.84)], and 120% for POPs [RR 2.2 (95%CI, 1.99–2.52)]. Six months after starting use of HCs, the risk of using antidepressants peaked by 40% [RR 1.4 (95%CI, 1.34–1.46)]. When the reference group was changed to those who never used hormonal contraception, the risk ratio estimates for users of COCs increased to 70% [RR 1.7 (95%CI, 1.66–1.71)].
de Wit et al. (29)	To investigate the association between oral contraceptive use and depressive symptoms, and examine whether this association is affected by age, and determine which specific symptoms are associated with oral contraceptive use.	A quantitative, prospective, population-based longitudinal cohort study.	Netherlands	AGYW aged 16 to 25 years, with age-stratified results for 16–19, 20–22, and 23–25 years.	1010	Depressive symptoms	Oral contraceptive pills (OCPs).	• For the overall cohort, OCP use was not associated with depressive symptoms (*β* = 0.006; 95%CI, –0.013 to 0.025; *p* = .52). • Age significantly interacted with OCP use (β = –0.021; 95% CI, –0.038 to –0.005; *p* = .0096) and this association was driven by differences in 16-year-old girls. • At this age, girls who used OCPs had higher concurrent depressive symptoms than their non-using counterparts (β = 0.075; 95% CI, 0.033–0.120; *p* < .001).
Zettermark et al. (64)	To investigate how the association between hormonal contraceptive (HC) use and antidepressant use.	Nationwide, quantitative, prospective, longitudinal cohort study.	Sweden	AGYW aged 12–30 years, with age-stratified results for 12–17, 18–23, and 24–30 years.	915,952	Use of antidepressants as a proxy for depression	Hormonal contraceptives (HCs)	• Among adolescents aged 12–17, HC use was consistently associated with higher odds of antidepressant use, regardless of mental health history, with ORs ranging from 1.1 (95% CI: 0.9–1.2) to 10.6 (95% CI: 1.4–19.9). • Among young adults aged 18–23, the association between HC use and antidepressant use was weaker and more variable, with an overall ORs ranging from 0.0 (95% CI: –0.2–0.1) to 8.8 (95% CI: 3.4–14.4). • The weakest association was especially among those aged 18–23 years without prior mental health issues, with ORs ranging from 0.0 (95% CI: –0.2–0.1) to 1.1 (95% CI: 0.5–1.8).
Moore et al. (17)	To examine the relation between mental health and dual contraceptive method use among sexually active college women at risk for STI and unintended pregnancy.	A quantitative, cross-sectional study.	United States	Young women aged 18–25 years.	307	Depression and perceived stress	Dual contraceptive methods (use of both HCs and barrier)	• A prior depressive disorder diagnosis was significantly associated with lower odds of dual method use compared to use of other contraceptive methods combined (aOR, 0.39; 95% CI: 0.19–0.79, *p* < 0.05), use of no method (aOR, 0.12; 95% CI: 0.03–0.55, *p* < 0.05), or use of hormonal contraceptives only (aOR, 0.39; 95% CI: 0.18 0.85, *p* < 0.05). • In contrast, stress was not significantly associated with dual method use compared to hormonal (aOR = 0.93, 95% CI: 0.50–1.75, *p* > 0.05), condom (aOR = 0.83, 95% CI: 0.38–1.81, *p* > 0.05), or no method use (aOR = 0.81, 95% CI: 0.27–2.43, *p* > 0.05).
Hall et al. (65)	To examine the influence of depressed mood and psychological stress on oral contraceptive (OC) side effects and discontinuation among young minority women.	A quantitative, secondary analysis of randomized control trial.	United States	AGYW aged 13–24 years	354	Depressed mood and psychological stress	HCs—oral contraceptives (OCs)	• At baseline, 21% experienced depressed mood and 19% stress, with 25% reporting mood changes and 57% weight changes at 6 months. • Only 38% continued with OCs at 6 months. Depressed mood (OR 2.27, *p* = .007) and stress (OR 2.07, *p* = .02) were associated with perceived OC-related moodiness. • Conversely, depressed mood (OR 0.54, *p* = .04), stress (OR 0.48, *p* = .03), and perceived weight change (OR 0.60, *p* = .03) decreased OC continuation likelihood at 6 months.
Steinberg et al. (66)	To examine how the association between both current and past depressive symptoms and the effectiveness level of the contraceptive method selected at a clinic visit varies by the type of reproductive health visit.	A quantitative, cohort sub-analysis, cluster-randomized trial.	United States	Young women aged 18 to 25 years.	1,215	Depressive symptoms	OCPs, transdermal patch, vaginal ring, injectable, natural family planning, emergency contraception, diaphragm, and condoms	• Women with current and past elevated depressive symptoms were more likely to use low vs. moderately effective contraceptive methods (RRR = 5.63, 95% CI = 2.31 to 13.71, *p* < .001). • However, current elevated depressive symptoms were not significantly associated with the choice between highly and moderately effective methods (RRR = 0.21, 95% CI = 0.02 to 2.12, *p* = .187).
Hall et al. (67)	To prospectively examine the influence of young women's depression and psychological stress symptoms on their weekly contraceptive method use.	A quantitative, prospective, longitudinal, population-based cohort study.	United States	Young women aged 18–20 years.	689	Depression and stress symptoms	LARC methods (LNG-IUD, injectable, implant), OCs, other hormonal methods—ring, patch, condoms, withdrawal, single method use, and dual method use	• Women with moderate to severe stress symptoms were over twice as likely not to use any contraceptive method (OR 2.23, CI 1.02–4.89, *p* = 0.04) compared to those without elevated stress. • Additionally, women with moderate/severe depression (RR 0.52, CI 0.40–0.68, *p* < 0.001) and stress (RR 0.75, CI 0.58–0.96, *p* = 0.02) symptoms had lower relative risks of using LARC methods than OCs. • Women with stress symptoms also had higher relative risks of using condoms (RR 1.17, CI 1.00–1.34, *p* = 0.02) and withdrawal (RR 1.29, CI 1.10–1.51, *p* = 0.001) than OCs
Rowlands et al. (13)	To examine the longitudinal associations between young women's physical and mental health and their method of contraceptive use over a 5-year period.	A quantitative, prospective longitudinal, population-based cohort study	Australia	Young women aged 18–23 years.	4,952	Psychological distress	OCPs. Barrier (Condoms) and LARC methods.	• Compared with women using the pill, women who used LARCs were more likely to report fair or poor general health (OR 1.50; 95% CI 1.28 to 1.76) and very high levels of psychological distress (OR 1.47; 95% CI 1.24 to 1.76). • Similar results were also found among women who used condoms were more likely to report fair or poor general health (OR 1.17; 95% CI 1.01 to 1.35) or very high levels of psychological distress (OR 1.34; 95% CI 1.13 to 1.60). • Non-users of contraception were also more likely to report very high levels of psychological distress (OR 1.49; 95% CI 1.24 to 1.78).
Toffol et al. (68)	To describe the sociodemographic, reproductive and mental health characteristics of all fertile-aged women who used hormonal contraception.	Nationwide, register-based, quantitative matched case-control study.	Finland	AGYW aged 15–49 years, with age-stratified results for 15–19, 20–24, 25–29, 30–34, 35–39, 40–44, 45–49 years.	588,712	Mood disorders, anxiety disorders, personality disorders, and intentional self-harm	Hormonal contraceptives-COPs and LNG-IUDs.	• Women with personality disorders were less likely to use HCs (0.89, 0.79 to 0.97, *p* < 0.05), with absolute risk differences between women with and without mental disorders ranging from 3.1% (anxiety disorders) to 10.1% (eating disorders). • Women aged 15–19 with recent substance abuse (*p* < 0.014) and anxiety disorder episodes *p* < 0.001 were significantly associated with HC use, but the effect was lower compared to other age groups
Lewandowski et al. (69)	To examine the association of hormone-based contraceptives with self-and parent-rated quality of life and mental health problems in girls aged 15–17 years.	Nationwide, quantitative, prospective, longitudinal cohort study.	Germany	Adolescent girls aged 15–17 years	1695	Mental health problems (Strengths, Difficulties) and Psychotropic drug use.	Hormonal contraceptives-(COPs)	• Although there was a trend of higher psychotropic drug prescriptions among OCP users, it was not statistically significant as compared to HC users (7.8% vs. 14.4%, *p* = 0.052). • OC use was not significantly associated with any indicators of mental well-being, regardless of whether they were self- or parent-reported. For self-rated SDQ scores, the association was non-significant [B = 0.312, 95% CI (−0.311, 0.935), *p* = .326], and similar findings were observed for parent-rated SDQ [B = 0.577, 95% CI (−0.053, 1.206), *p* = .073]. • Self-reported KINDL-R scores also showed no significant association [B = −0.145, 95% CI (−1.656, 1.366), *p* = .850]. Likewise, parent-rated KINDL-R scores were not significant [B = 0.038, 95% CI (−1.429, 1.506), *p* = .959].
Harper et al. (3)	To assess the association between symptoms of depression, anxiety, and stress with delays in getting a contraceptive method or prescription among older adolescents and young adults.	A quantitative, prospective longitudinal study.	United States	AGYW aged 18–29 years, with age-stratified results for 18–19 and 20–29 years.	1,665	Depression, anxiety, and stress symptoms	HC methods and Barrier methods (condoms).	• Participants with elevated depression symptoms were 1.58 times more likely to delay contraception than those without [aOR 1.58, 95% CI (1.27–1.96), *p* < 0.001]. • Similarly, those with elevated anxiety and stress symptoms had 1.46 times higher odds of delaying contraception [aOR 1.46, 95% CI (1.17–1.82), *p* < 0.001]. • Additionally, Adolescent participants aged 18–19 years were significantly more likely to delay getting contraception they thought they needed compared with young adults (aOR 1.32, 95% CI 1.07–1.63, *p* < 0.05).
Lundin et al. (35)	To investigate the association between hormonal contraceptive (HC) use and depression, measured as antidepressant treatment or a first depression diagnosis subsequent to HC use.	A quantitative, retrospective, register-based, longitudinal cohort study	Sweden	AGYW aged 15–25 years	739,585	Depression	COCs, POPs, non-oral combined products—patch and vaginal ring. Non-oral POPs, injectable, implant, and LNG-IUD.	• COC use in adolescent girls aged 15–19 years conferred no risk increase of depression (RR 0.96, 95% CI 0.93–0.98). • In contrast, the used of oral POPs (RR 1.13, 95% CI 1.07–1.19), contraceptive patch/vaginal ring (RR 1.43, 95% CI 1.30–1.58), implant (RR 1.38, 95% CI 1.30–1.45), and LNG-IUD (RR 1.58, 95% CI 1.48–1.68) were associated with higher risk of depression in adolescents. • Among women 20–24 years of age, use of COC (RR 0.80, 95% CI 0.78–0.82) and oral POPs (RR 0.96, 95% CI 0.92–0.99) was associated with a decreased risk, whereas use of any non-oral HC was associated with slightly increased risks.

HCs, hormonal contraceptives; CHCs, combined hormonal contraceptives; COCs, combined oral contraceptives; POPs, progestin-only pills; OCs, oral contraceptives; OCPs, oral contraceptive pills; LNG-IUD, levonorgestrel intrauterine device; LARC, long-acting reversible contraceptives; AGYW, adolescent girls and young women.

#### 2.1.5 Collating, summarizing, and reporting the results

After data charting, the synthesis process began by systematically organizing the content to ensure that the data were consistent and prepared for subsequent analysis (70). Quantitative content analysis (QCA) was employed to synthesize and analyze data extracted from the included studies, and this analysis method is defined as a systematic and replicable method of categorizing and interpreting content to quantify patterns in study findings (71). As outlined by Huxley (71), the studies were subsequently organized into broader categorical variables to enable a systematic and coherent synthesis of the findings. The categories were then analyzed to identify relationships and recurring patterns across the studies, with a particular emphasis on frequencies as a means of quantifying trends. Visual formats such as graphs, charts, and tables were used to represent these findings for accessibility and clarity. The final step involved reporting, where results were narrated in a structured format to explain key insights and implications. This systematic approach of integrating QCA and narrative reporting ensured rigor and a robust framework for highlighting similarities, differences, and overall patterns in the data by synthesizing it into actionable insights (70, 71).

## 3 Results

### 3.1 Characteristics of the included studies

**Figure 2:**
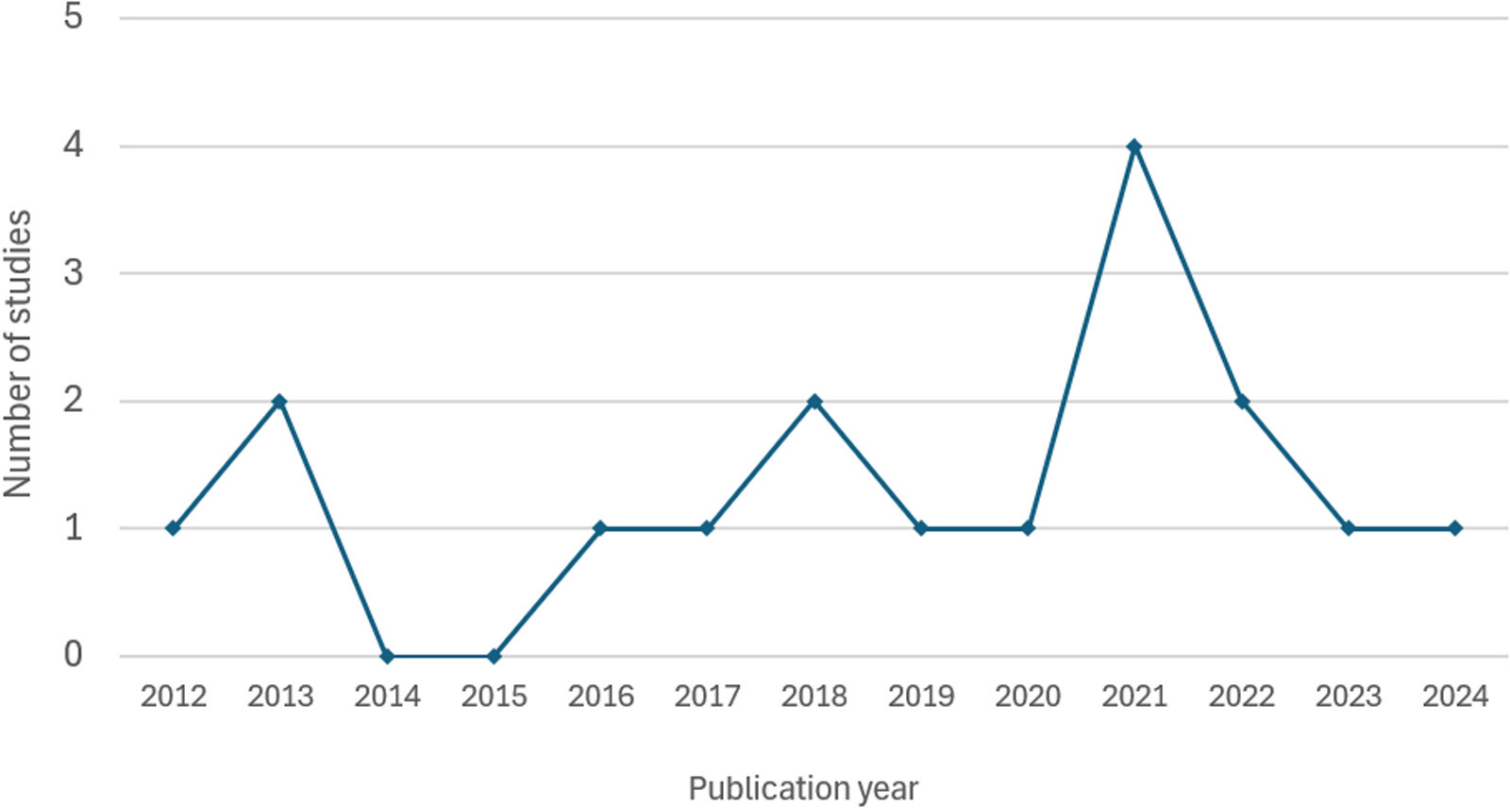
Distribution of the number of studies published by year (*N* = 17).

Table 2 summarizes the characteristics of each of the 17 included studies. These studies were published between 2012 and 2024, with sample sizes ranging from 178–1,061,997. Figure 2 demonstrates the distribution of the number of studies published annually during this period. One study was published in 2012 (65), followed by a slight increase in 2013 with two studies (63, 67). No studies were published in 2014 and 2015, but 2016 (30), 2017 (17), 2019 (29), and 2020 (69) each year had at least one study published, while 2018 had two published studies (59, 66). The year 2021 was marked by the highest outputs, with four published studies (13, 35, 60, 64). However, this slight peak was followed by a decline, with only two studies published in 2022 (61, 68) and a further drop to one study published in 2023 (62) and 2024 (3) (see Figure 2).

**Figure 3:**
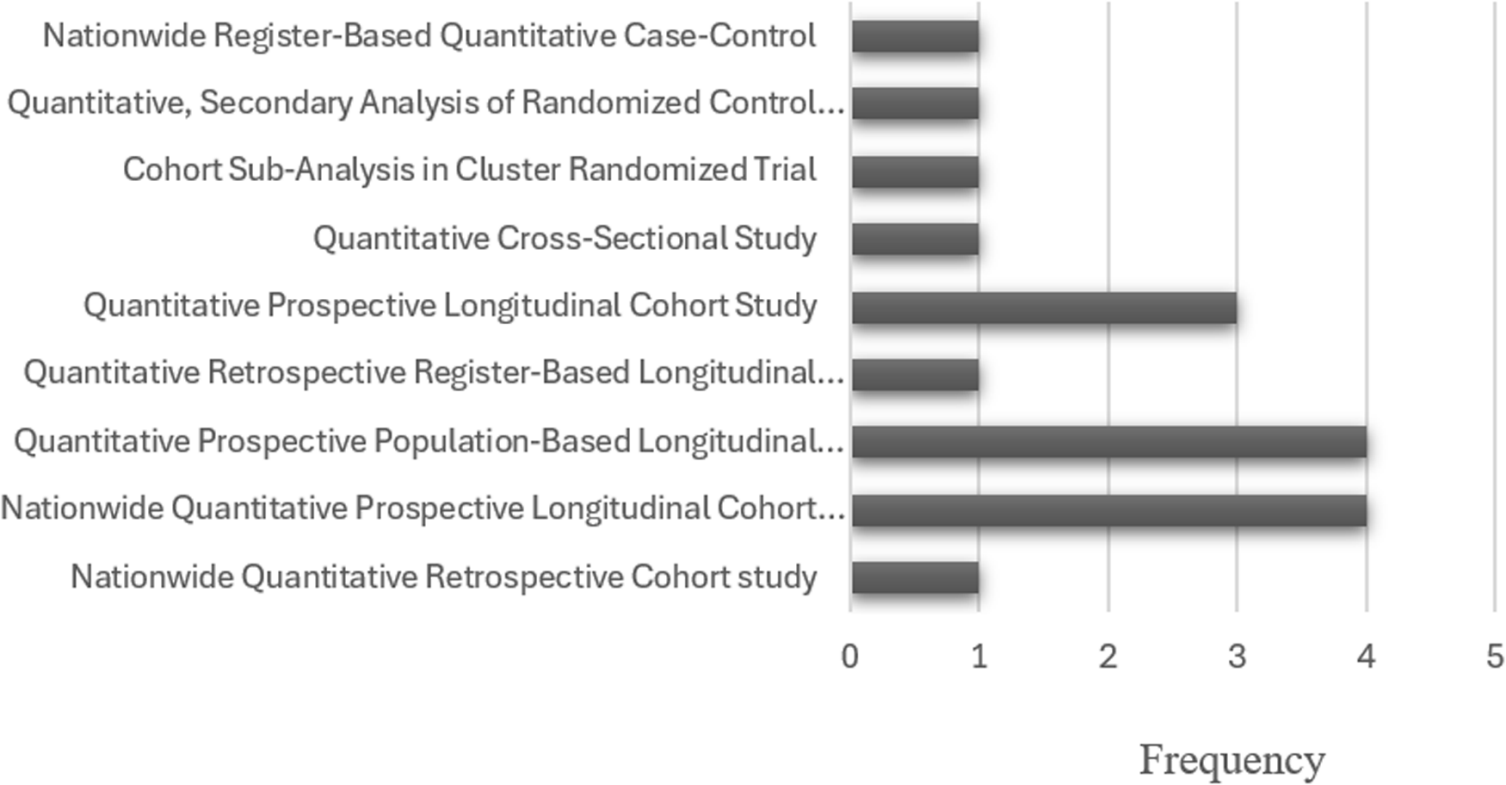
Frequency of study approaches and designs in the included studies (*N* = 17).

Figure 3 presents an overview of the study approaches and designs used in the included studies. All the included studies adopted a quantitative approach, but the specific study designs were varied across the studies. Most studies (76%; *n* = 13) used longitudinal cohort designs (3, 13, 29, 30, 35, 59, 61–64, 67, 69). Four were nationwide prospective longitudinal cohort studies (30, 62, 64, 69). There were also four prospective population-based cohort studies (13, 29, 60, 67). Three studies were prospective longitudinal cohort studies (3, 61, 63). One was a nationwide retrospective cohort study (59), and one retrospective register-based cohort study (35). Studies used different designs, including one cross-sectional study (17), one cohort sub-analysis in a cluster randomized trial (66), one secondary analysis of a randomized control trial (65), and one nationwide register-based matched case-control study (68).

**Figure 4:**
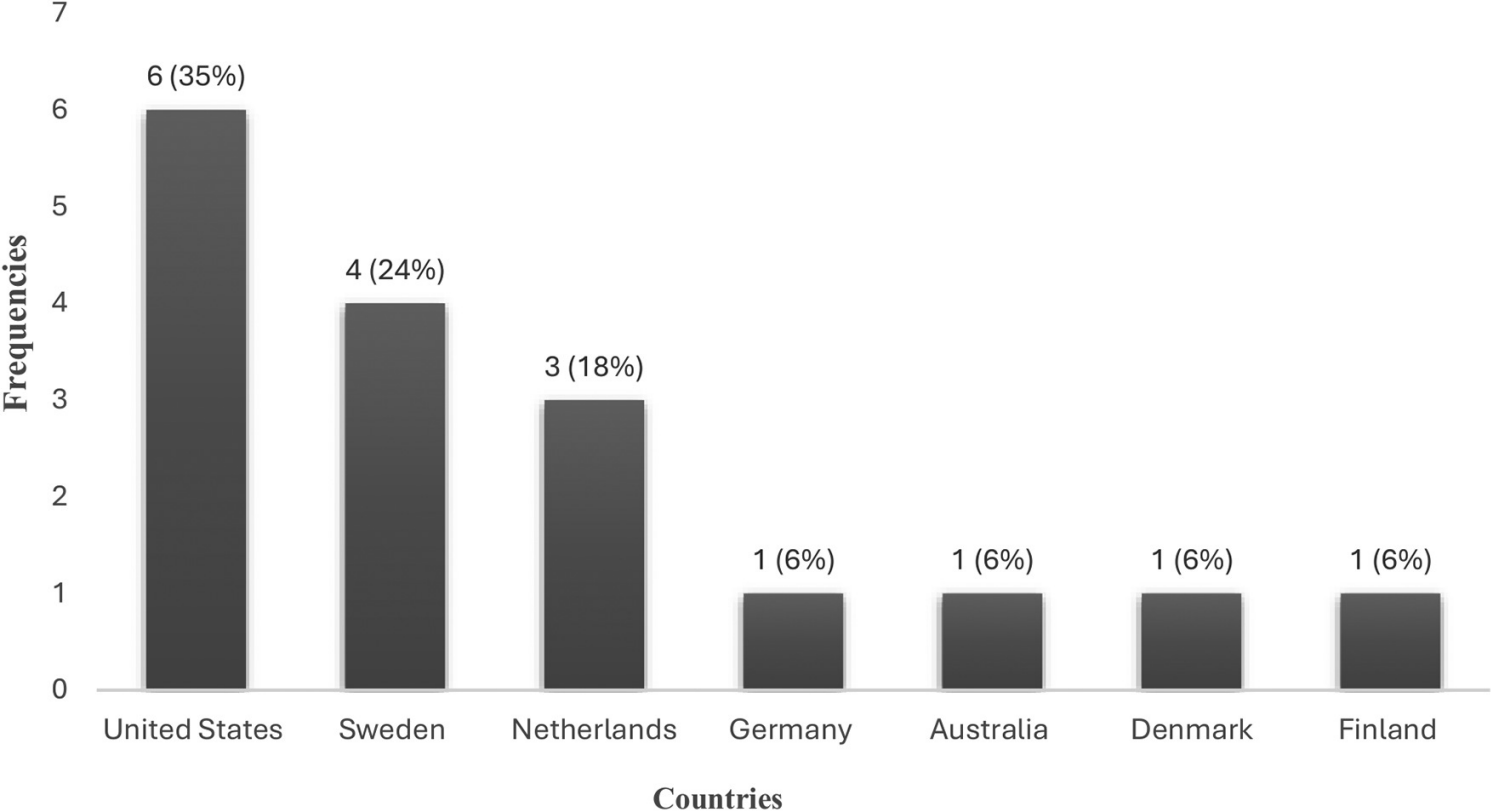
The geographical distribution of the included studies by country (*N* = 17).

Figure 4 depicts the geographical distribution of the included studies by country. Most studies (35%; *n* = 6) were conducted in the United States (3, 17, 63, 65–67). Sweden followed with four studies (35, 59, 62, 64), while the Netherlands contributed three studies (29, 60, 61). Other countries, such as Denmark (30), Australia (13), Finland (68), and Germany (69) were each represented by only one study.

**Figure 5:**
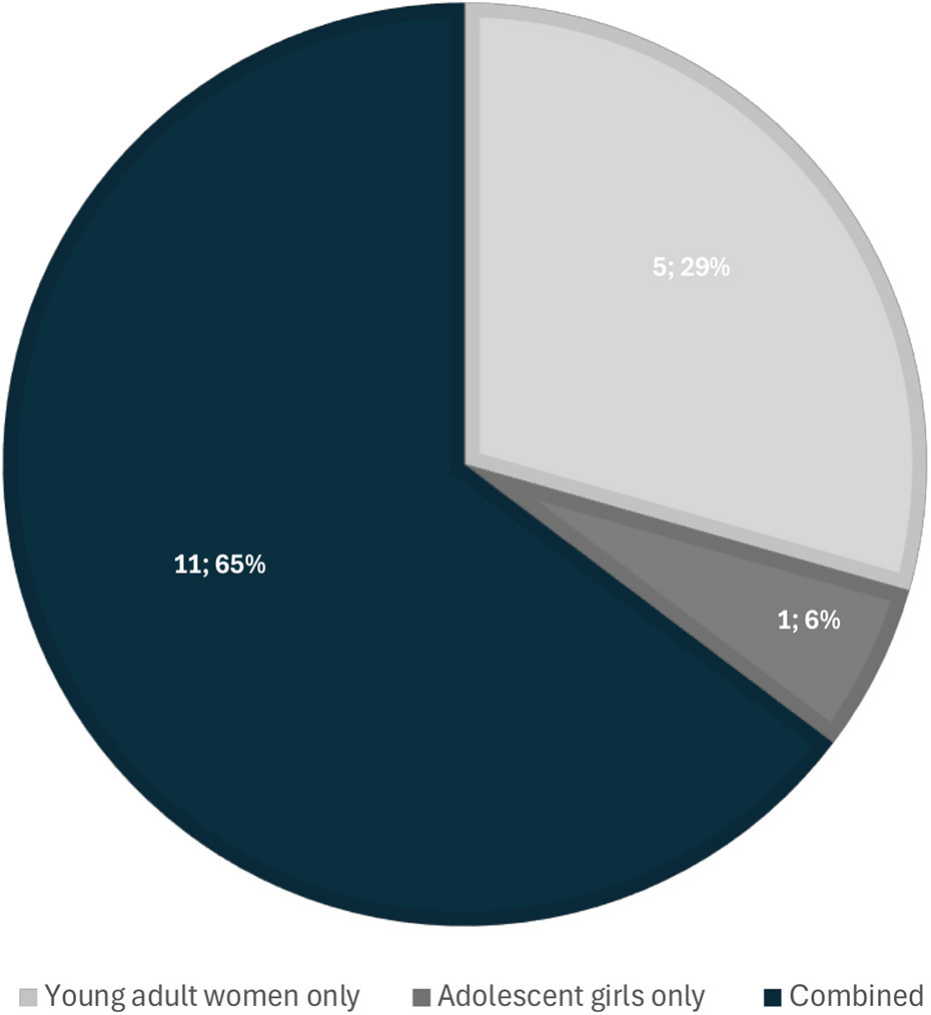
Representation of adolescents (≤19 years), young women (20–25 years), or both among the included studies. (*N* = 17).

Figure 5 depicts representation of adolescents (≤19 years), young women (20–24 years), or both across included studies. Most studies (*n* = 11; 65%) included both AGYW (3, 29, 30, 35, 59–62, 64, 65, 68). Five studies included data for young adult women participants but not adolescents (13, 17, 63, 66, 67), and one study included only adolescents (69).

### 3.2 Summary of results of the analyzed studies

#### 3.2.1 Mental health factors

Depression (*n* = 11; 65%) was the most frequently studied factor (3, 17, 29, 35, 60–63, 65–67). Stress (*n* = 5; 29%) followed by being the second most explored factor (3, 17, 63, 65, 67), while proxies for mental distress were studied in four studies (30, 59, 64, 69). Anxiety was examined in three studies (3, 61, 68), whereas psychological distress (13), mood disorders (68), and other mental health problems (69) were each investigated in only one study (see Table 3).

**Table 3:** Mental health factors, direction of associations, and contraceptive types.

Categories	Frequency (*n*)	Proportion (%)
Mental health factors		
Depression	11 (3, 17, 29, 35, 60–63, 65–67)	65%
Anxiety	3 (3, 61, 68)	18%
Proxies for mental distress	4 (30, 59, 64, 69)	24%
Stress	5 (3, 17, 63, 65, 67)	29%
Psychological distress	1 (13)	6%
Mood disorder	1 (68)	6%
Other mental health factors	1 (69)	6%
Contraceptive types		
Hormonal contraceptive methods	17 (3, 13, 17, 29, 30, 35, 59–69)	100%
Dual contraceptive methods	2 (17, 67)	12%
Barrier contraceptive methods	5 (3, 13, 63, 66, 67)	29%
Traditional methods	2 (66, 67)	12%
Direction of associations		
Positive association	12 (3, 13, 30, 35, 59–62, 64–67)	71%
Negative association	5 (17, 63, 65, 67, 68)	29%
Non-significant/ No association	5 (17, 29, 35, 66, 69)	29%

#### 3.2.2 Contraceptive types

HC was the most studied method, with all studies (100%; *n* = 17) examining this method (see Table 3). Barrier contraceptives (29%; *n* = 5) were the second most examined method (3, 13, 63, 66, 67). Dual contraceptive use (17, 67) and traditional (66, 67) contraceptives were the least studied methods, with two studies for each (see Table 3).

### 3.3 Direction of associations between mental health factors and contraceptive use

Table 3 also presents an overview of the direction of associations found from the results of the included studies. Twelve studies (71%) found that the mental health conditions and symptoms were positively associated with contraceptive use (3, 13, 30, 35, 59–62, 64–67). Five studies (29%) indicated that mental health symptoms were negatively associated with effective contraceptive use (17, 63, 65, 67, 68). Another five studies (29%) reported non-statistically significant or no associations between mental health symptoms and contraceptive use (17, 29, 35, 66, 69) (see Table 3).

#### 3.3.1 Positive associations between mental health factors and contraceptive use

A total of 12 studies (71%) found that mental health conditions and symptoms were associated with increased odds of not using the contraceptives (3, 13, 30, 35, 59–62, 64–67). Five of these studies showed that oral contraceptives such as COCs and progestogen-only pills were associated with an increased risk of depression, while non-OCs such as LNG-IUDs, patches, and vaginal rings also showed a higher risk of depression (30, 35, 60, 62, 65). Three of these studies found that the risk of depression was higher in younger users of non-oral contraceptives who used these methods for the first time (30, 35, 62) (see Table 2).

Two studies found that HC use, such as non-oral progesterone-only methods, was strongly associated with increased psychotropic drug and antidepressant use, with the highest risk observed in younger adolescents compared to non-users (59, 64). Two studies found that adolescent OC users had stable anxiety symptoms, in contrast to non-users who experienced increased anxiety in late adolescence, with elevated anxiety symptoms linked to the higher odds of delayed contraceptive use (3, 61). Of these two studies, Doornweerd et al. (61) revealed a protective effect of OC users who showed a stable trajectory of anxiety symptoms compared to non-users. On the contrary, Toffol et al. (68) found that women aged 15–19 years with recent episodes of anxiety disorder had higher odds of HC use in contrast to those without these conditions (see Table 2).

Only one study found that the users of LARC methods were associated with a higher risk of psychological distress (13) (see Table 3). Two studies found the influence of stress on contraceptive use, where women with moderate to severe stress symptoms were less likely to use any HC method and more likely to opt for barrier methods (3, 67) (see Table 2).

#### 3.3.2 Inverse and no association between mental health factors and contraceptive use

Of five studies that found an inverse association, three studies identified depression, whether moderate to severe, depressed moods, or a lifetime history of it to be a significant factor in reducing odds of contraceptive use, particularly with methods that require consistent use, such as LARCs, OCs and dual contraceptive methods (17, 65, 67). Specifically, Hall et al. (67) found that women with moderate to severe depression had a lower risk of using LARC methods than OC users, while Moore et al. (17) revealed that a lifetime history of depression was associated with significantly lower odds of using dual contraceptive methods compared to non-users and HC users only. Similarly, Hall et al. (65) found that depressed mood reduced the likelihood of OC continuation at six months.

Additionally, three studies demonstrated that stress was consistently associated with lower odds of using contraceptive methods (63, 65, 67). Specifically, Hall et al. (65) found that stress significantly reduced the odds of OC continuation at six months. In their first study, Hall et al. (67) reported that stress was associated with lower odds of using LARC methods. In their second study, published in the same year, Hall et al. (63) found that stress symptoms were associated with inconsistent use of contraceptive methods such as OCs, condoms, and withdrawals. Regarding anxiety, one study by Toffol et al. (68) identified an absolute risk difference in the likelihood of not using HCs among women with anxiety disorders. In the episodes of other mental health factors, Toffol et al. (68) found that women with personality disorders had lower odds of using HCs.

In addition, of the five studies that reported no association between mental health factors and contraceptive use, two found no significant association between depressive symptoms and HC use, including OCs and COCs (29, 35). For instance, Lundin et al. (35) found that COC use was not associated with an increased risk of depression, even among adolescents aged 15–19 years. de Wit et al. (29) also reported no significant association between OC use and depressive symptoms across all age groups. One study found that stress was not significantly associated with dual contraceptive use (17). Another study by Lewandowski et al. (69) found no significant differences in mental health outcomes, such as general mental distress and psychotropic drug use, between OC users and non-users. To end, Steinberg et al. (66) also found no significant association between elevated depressive symptoms and the use of highly or moderately effective contraceptive methods (See Table 3).

### 3.4 Bidirectional influences between mental health factors and contraceptive use across age groups

Table 4 presents results that were grouped by directionality (whether mental health factors influenced contraceptive use or vice versa) and age (adolescents: 10–19 years vs. young adults: 20–25 years). To ensure age–based comparisons, three studies (13, 17, 61) that included overlapping age bands in their results (e.g., 15–25 or 18–25) were excluded for clarity from this age-stratified synthesis.

**Table 4:** Bidirectional influences between mental health factors and contraceptive use across age groups.

Age Group	Bidirectionality	Number of Studies	Significance of the results (<0.05)
Adolescents (10–19 years)	Mental health factors → Contraceptive use (3, 65, 67, 68)	4	Yes
	Mental health factors ← Contraceptive use (29, 30, 35, 59, 62, 64, 69)	7	Mixed
Young Adults (20–25 years)	Mental health factors → Contraceptive use (63, 66)	2	Yes
	Mental health factors ← Contraceptive use (30, 35, 60, 62)	4	Yes

#### 3.4.1 Adolescents (10–19 years)

Four studies (3, 65, 67, 68) reported that mental health factors influenced contraceptive use among adolescents. For example, Hall et al. (65) found that stress and depressed mood significantly reduced OC continuation. In another study, Hall et al. (67) reported that depression and stress significantly reduced LARC use, while stress increased reliance on condoms and withdrawal. Toffol et al. (68) reported that personality and anxiety disorders were significantly associated with reduced HC use among adolescents. Harper et al. (3) found that adolescents aged 18–19 years with depressive symptoms, stress, and anxiety were significantly more likely to delay obtaining contraception compared to young adults. (see Tables 2, 4).

Seven studies (29, 30, 35, 59, 62, 64, 69) demonstrated that contraceptive use influenced mental health outcomes in adolescents. For instance, Zettermark et al. (59) found significant associations between HC use and psychotropic medication use, especially with non-oral progesterone methods. Skovlund et al. (30) identified a significant increase in antidepressant use among adolescent users of COCs and POPs. Stenhammar et al. (62) reported a significant increase in depression risk with LNG-IUD use before the age of 20 years. Lundin et al. (35) found a significant increase in depression risk with non-oral HCs but no risk with COCs. de Wit et al. (29) found that age significantly interacted with OCP use on depressive symptoms, with the association driven by differences in 16–19–year–old girls. Zettermark et al. (64) found that among adolescents aged 12–17, HC use was consistently and significantly associated with higher odds of antidepressant use, regardless of mental health history. On the contrary, Lewandowski et al. (69) found no significant associations between OC use and mental health outcomes in girls aged 15–17 (see Tables 2, 4).

#### 3.4.2 Young adults (20–25 years)

Two studies (63, 66) reported that mental health factors significantly influenced contraceptive use among young adults. Hall et al. (63) found that depression and stress symptoms significantly reduced weekly consistency in contraceptive use. Steinberg et al. (66) noted a tendency toward selection of less effective methods among women with depressive symptoms (See Tables 2, 4).

Four studies (30, 35, 60, 62) indicated that contraceptive use significantly influenced mental health outcomes. Anderl et al. (60) found that adolescent OC use significantly predicted a higher risk of major depressive disorder in early adulthood. Stenhammar et al. (62) reported a significant increase in risk of depression associated with LNG-IUD use among women aged 20–24 years. Skovlund et al. (30) found significant associations between COC, POP, LNG-IUD, patches, vaginal rings use, and antidepressant use in women aged 20–24 years. In contrast, Lundin et al. (35) found a significantly reduced depression risk with COC and POP users compared with non-OC users, although the effect sizes were smaller (See Tables 2, 4).

## 4 Discussion

The review study aimed to map and synthesize the available peer–reviewed literature to understand how mental health conditions and symptoms influence contraceptive use among AGYW globally.

The findings highlight an overrepresentation of longitudinal cohort studies. This pattern indicates a substantial gap in research that examines the temporary influence of mental health conditions and symptoms on contraceptive behavior. Comparing these findings with Kraft et al.'s (18) systematic review, which focused on the symptoms of mental disorders and OC use, both studies emphasize the prevalence of longitudinal cohort designs. However, Kraft et al. (18) incorporated a wide array of studies, including randomized controlled trials and cross–sectional studies, which were less common in our review. In contrast, a scoping review conducted by Odette et al. (72) on the effects of depression and anxiety on non–barrier contraception among Adolescents and Young Adults primarily found that most of the studies were cross–sectional. This methodological variability underscores the range of designs that have been applied in this research area over time, but it also points to a paucity of the use of other causal designs, such as experimental, cross–lagged panel, quasi-experimental, and natural experiment. These designs may provide robust evidence regarding the immediate effects of and causal relationships between mental health factors and contraceptive use. Regarding geographic distribution, there is a clear emphasis on high–income countries such as Sweden, Denmark, the Netherlands, Germany, the United States, Finland, and Australia (73). This aligns with the findings from Kraft et al. (18) and Odette et al. (72) review studies. This consistent trend may suggest that data availability and research resources in these countries are well developed, while it also highlights the paucity of studies from LMICs.

Additionally, across various settings, in LMICs AGYW often face unique challenges that are influenced by the socio-cultural and economic factors associated with mental health and contraceptive behavior (74, 75). The geographical imbalances identified in terms of study settings suggest the lack of global applicability of findings in other regions where socio–cultural, political, and economic factors shape contraceptive behavior in unique ways (74, 76). Further research should span across diverse regions of the world with a particular focus on LMICs. This would ensure a more nuanced understanding of how mental health conditions and symptoms affect contraceptive use among AGYW across different contexts. Regarding sample characteristics, the current review includes studies that focused on both AGYW, but with some studies focusing either on young women or adolescent girls. Notably, one study conducted by Lewandowski et al. (69) focused exclusively on adolescent girls, which suggests a significant gap in the literature that focuses solely on the adolescent population. A similar trend was reported by Odette et al. (72), where most studies included both AYA, while two studies included only adolescents. The limited number of studies focusing exclusively on adolescents obscures the unique challenges they encounter regarding mental health and contraceptive use during this critical developmental stage. While research on both age groups is crucial, more adolescent–focused studies are needed to better understand their unique mental health dynamics in the context of contraceptive use.

Our review corroborates the prevalence of depressive symptoms as the most frequently studied (*n* = 11) mental health factor that affects contraceptive use. Our findings correlate with both Odette et al.'s (72) and Kraft et al.'s (18) reviews reported that most studies (*n* = 14, respectively) focused on depressive symptoms. Although anxiety and mood disorders were less frequently examined in the reviews by Odette et al. (72) and Kraft et al. (18), the present review identified stress, anxiety, psychological distress, and mood disorders as key mental health conditions. These findings collectively underscore the complex role of mental health factors in the context of contraceptive use. This suggests that while Kraft et al. (18) and Odette et al. (72) primarily identified depression as the most studied mental health factor, the current review provides a broader perspective on the full spectrum of mental health factors influencing contraceptive use. Regarding the types of contraceptive methods, our findings align with those of Odette et al. (72), as both reviews identify HCs as the most frequently studied methods, while research on dual contraceptive methods remains limited. Therefore, the dearth of studies on dual contraceptive use indicates a substantial gap in gaining a comprehensive understanding of methods that combine HCs with barrier methods for enhanced protection (72, 77).

The studies that were reviewed highlight three main ways in which mental health conditions and symptoms influence contraceptive use. Firstly, a significant number of studies found a positive association between mental health conditions and symptoms, such as depression and anxiety, major depressive disorders and anxiety disorder, and the use of contraception. Odette et al. (72) highlighted that in 16 of the 18 reviewed studies, depression and anxiety were positively associated with inconsistent contraceptive use, including lower odds of dual contraceptive use. Similarly, our review found that twelve studies (71%) reported a positive association between mental health factors and contraceptive use, with HCs being associated with an increased risk of depression, particularly among younger users (60, 62). This raises concerns about the psychological side effects of HCs and their influence on consistent use among AGYW (78). However, some studies suggest a stable effect of anxiety symptoms among OC users compared to non–users who experience high levels of anxiety in late adolescence (3, 61). This indicates that for certain groups of adolescents, HCs may provide psychological benefits, such as stabilizing anxiety levels. However, the relationship between HC use and depression involves a mutual influence, which remains a major threat to many AGYW's mental health.

On the other hand, a subset of studies found an inverse association, where mental disorders were associated with lower odds of contraceptive use. Four studies in our review reported that mental disorders, particularly depression, led to lower odds of contraceptive use. For instance, Hall et al. (67) found that women with moderate to severe depression were less likely to use LARCs or OCs, which was indicative of how severe depressive symptoms can affect consistent and effective use of contraceptives. Similarly, stress was consistently associated with lower odds of contraceptive use, especially with methods that demanded optimal adherence (17, 65). This suggests that mental health challenges, such as low motivation, cognitive difficulties, and emotional instabilities, can interfere substantially with contraceptive uptake and use. Several studies, however, reported no significant associations between mental health conditions and contraceptive use. For example, two studies reported no association between depressive symptoms and HC use, including OCs and COCs (29, 35).

For instance, Lundin et al. (35) reported that COC use was not significantly associated with an increased risk of depression, even among adolescents aged 15–19 years. Similarly, De Wit et al. (29) found no significant association between OCP use and depressive symptoms, and this finding was contrary to the results found by Anderl et al. (60) and Stenhammar et al. (62) that underscored a significant association between these variables. However, de Wits (29) yielded more nuanced findings whereby age emerged as a significant moderator in the otherwise non-significant association between OCP use and depressive symptoms, specifically among 16-year-old girls using OCPs. This suggests that the impact of OCPs on psychological well-being may be developmentally contingent, highlighting the significance of considering age and psychological vulnerability when initiating OCP use during adolescence. Moore et al. (17) found that stress was not significantly associated with dual contraceptive use, while Lewandowski et al. (69) reported no significant differences in mental health outcomes between OC users and non-users. Likewise, Steinberg et al. (66) found no significant association between elevated depressive symptoms and the use of highly or moderately effective contraceptive methods. These findings align with Kraft et al.'s (18) research, which identified that most studies found no association between symptoms of mental disorders and OC use.

Given the significant variations in the results, bidirectional relationships were assessed and unfolded differently across age groups to deepen our understanding of both mutual influences and age-specific nuances. Among adolescents (10–19 years), mental health symptoms such as stress, depression, anxiety, and certain personality disorders were consistently associated with disrupted contraceptive behaviors, including lower continuation rates and delayed uptake (3, 65, 67, 68). These findings suggest that adolescents may lack the emotional regulation, autonomy, or access required to maintain consistent contraceptive use under conditions of psychological distress, which may be due to their developmental stage and social dynamics. In contrast, among young adults (20–25 years), although fewer studies explored the influence of mental health on contraceptive behaviors, depressive symptoms still appeared to reduce consistency and shift preferences toward less effective methods (63, 66). This suggests that while young adults may have greater reproductive autonomy compared to adolescents, psychological and emotional vulnerabilities still play a crucial role in shaping their adherence and decisions regarding contraceptive use.

The reverse direction of influence, whereby contraceptive use affects mental health, also showed variation across age groups. Among adolescents, HC use was more frequently and strongly associated with increased risks of depressive symptoms and psychotropic medication use (29, 30, 35, 59, 62, 64), particularly with both oral and non-oral methods. This suggests that adolescents have high biological and psychosocial sensitivity to hormonal changes emanating from the use of contraceptives. However, results were not consistent, with Lewandowski et al. (69) reporting no associations, indicating that methodological differences (HC type, contexts, time, mental health measure) may contribute to conflicting findings. Among young adults, evidence also supported associations between contraceptive use and depressive outcomes (30, 35, 60, 62), but the effects were often smaller or more varied than in adolescents. For instance, while some studies showed increased depression risk (30, 60, 62), Lundin et al. (35) found reduced risk with specific methods, such as COCs and POPs, suggesting that individual-level factors such as mental health and contraceptive history, or context, might confound the outcomes in this age group.

Overall, these findings imply that mental health considerations are critical in contraceptive counseling, but with differentiated services by age. For adolescents, there is a pressing need for integrated services that address psychological and emotional well-being in conjunction with contraceptive provision, particularly for those initiating hormonal methods. For young adults, tailored services and support should recognize the persistence of influences of mental health factors but also the increasing complexity of contraceptive decision-making at this life stage.

### 4.1 Implications

This review has significant implications for research, practice, and policy. For research, the findings emphasize the need for methodological diversity, including experimental, correlational, qualitative, and mixed methods studies to explore causal pathways, contextual influences, and lived experiences. Specifically, prospective studies are needed to investigate temporary influence, while qualitative research can provide in-depth descriptions of psychosocial and emotional dimensions of contraceptive experiences. Future research should also bridge the gaps in dual and non-HC methods by extending the focus beyond HCs and high-income settings. Additionally, research should explore individual trajectories by investigating how perceived agency, age, and psychosocial environments intersect with age to shape both mental health and contraceptive experiences of AGYW, especially in LMICs. In practice, healthcare professionals should systematically integrate mental health screening into contraceptive counselling sessions to provide age and method-specific support and services holistically. Training frontline providers in mental health literacy and referral pathways can enable early identification and management of distress, thereby addressing issues related to contraceptive use and unintended pregnancies. At the policy level, the findings underscore the need for cross–sectional policy frameworks that explicitly integrate mental health services into SRH programs. Additionally, policymakers must prioritize coordinated and sustained access to both mental health support and contraceptive services, particularly for AGYW experiencing psychological distress or diagnosed conditions. The development of national SRH strategies that mandate integration of differentiated services, fund community-based outreach, and monitor intersectional outcomes can enhance both effectiveness and accessibility of mental health and SRH services. Such integrative approaches may improve service uptake and inconsistent use of contraceptives to reduce the risk of unintended pregnancies in these vulnerable subsets of the population.

### 4.2 Strengths and limitations

There are various strengths in this review. It synthesizes results from different studies across several countries, which provides a broad perspective on the impact of mental health conditions and symptoms on contraceptive use. The inclusion of longitudinal cohort studies strengthened the findings by showing patterns over time. Additionally, the multifactorial aspect of mental health factors added breadth to the understanding of their impact on contraceptive behaviors. However, certain limitations exist. This review was limited to English-language publications due to resource constraints, including the need to ensure consistency, the time-intensive nature of professional translation services, and the additional effort required to validate non–English texts. The authors duly acknowledge that restricting publications to the English language only may underrepresent evidence from non-English-speaking regions, particularly LMICs. The overrepresentation of studies from high–income nations can limit the generalizability of findings to LMICs, which are most often affected by high rates of unintended pregnancy. The reliance on quantitative methods may also overlook valuable qualitative insights regarding the influence of mental health conditions and symptoms on contraceptive behaviors. Another limitation is the predominant focus of studies on HC methods, which limits the generalizability of findings related to the influence of mental health factors across the broader spectrum of contraceptive options, including the use of dual and barrier methods. Moreover, inconsistencies in the measurement of mental health conditions and symptoms and contraceptive use may affect the comparability across studies. Most studies did not adjust for potential confounders such as socioeconomic status, trauma history, or access to healthcare, which may have explained the observed associations. Given that the sixth–stage consultation exercise in Arksey and O'Malley's framework has been regarded as optional (79), and it was not included in this review due to the primary aim of mapping existing evidence and constraints related to time and resources. However, authors recommend that future scoping review studies should endeavor to embed knowledge users' engagement throughout the process and move towards a co–knowledge creation model (80). Additionally, this scoping review did not include a formal risk of bias assessment, as its purpose was mainly to map existing evidence rather than critically appraise the quality of the included studies. It lays the groundwork for future systematic reviews, which may narrowly focus on specific contraceptive method use and include applying critical appraisal and meta–analyses with pooled effect sizes to evaluate more in–depth the statistically significant outcomes. Therefore, the findings of this review should be interpreted with caution due to regional skewness, methodological constraints, and the limited coverage of the full range of contraceptive methods in the existing evidence.

## 5 Conclusion

The findings of this review provide considerable insights into the relationship between mental conditions and symptoms and the use of contraceptives among adolescent girls and young women (AGYW). While depression is the most frequently studied factor, it is critical to provide greater focus on other mental health conditions and symptoms, such as anxiety disorders, stress, and psychological distress. The review reveals a predominant focus on hormonal contraceptives, while non–HC methods, such as condoms, copper–IUD, diaphragm, and fertility awareness-based methods, received considerably less attention. This imbalance underscores a critical gap in the literature and limits the extent to which conclusions can be widely generalized regarding the influence of mental health conditions and symptoms across the full range of contraceptive options, including dual contraceptive use. The evidence from reviewed studies highlights the varying effects of mental health factors on contraceptive use, with several studies suggesting that symptoms of depression, anxiety, or stress reduced or delayed the odds of contraceptive uptake and use. Conversely, some evidence suggests that while the use of certain HCs, particularly non–oral methods, increased the risk of depressive symptoms among younger users, OCs showed a protective effect by demonstrating a more stable trajectory of anxiety symptoms compared to non-users. These mixed findings reflect that mental health effects may vary by method and age, and such variations underscore the need to consider individual and contextual factors in both mental health and contraceptive research. Additionally, they reinforce the importance of differentiated services that integrate mental health support with reproductive health care across developmental stages. Future research should adopt an integrated approach that includes qualitative findings to enhance understanding of the lived experiences of AGYW and the nuanced ways in which mental health conditions and symptoms affect their contraceptive uptake and use. Furthermore, research should primarily focus on LMICs to provide evidence that is globally representative and can effectively inform policies and interventions even in resource–poor settings. Policies and interventions aimed at improving contraceptive uptake, use, and adherence should prioritize the integration of mental health support and services to ensure holistic reproductive healthcare for AGYW.

## Data Availability

The data and findings presented in this study are accessible within the article. Any further inquiries can be addressed to the corresponding author.
